# Nitric oxide, interorganelle communication, and energy flow: a novel route to slow aging

**DOI:** 10.3389/fcell.2015.00006

**Published:** 2015-02-06

**Authors:** Alessandra Valerio, Enzo Nisoli

**Affiliations:** ^1^Department of Molecular and Translational Medicine, University of BresciaBrescia, Italy; ^2^Department of Medical Biotechnology and Translational Medicine, Center for Study and Research on Obesity, University of MilanMilan, Italy

**Keywords:** mitochondrial biogenesis, peroxisomes, endoplasmic reticulum, aging, nitric oxide, endothelial NO synthase (eNOS), reactive oxygen species, hormesis

## Abstract

The mitochondrial lifecycle (mitochondrial biogenesis, dynamics, and removal by mitophagy) is carefully orchestrated to ensure the efficient generation of cellular energy and to maintain reactive oxygen species (ROS) production within an optimal range for cellular health. Based on latest research, these processes largely depend on mitochondrial interactions with other cell organelles, so that the ER- and peroxisome-mitochondrial connections might intervene in the control of cellular energy flow. Damaged organelles are cleared by autophagic mechanisms to assure the quality and proper function of the intracellular organelle pool. Nitric oxide (NO) generated through the endothelial nitric oxide synthase (eNOS) acts a gas signaling mediator to promote mitochondrial biogenesis and bioenergetics, with a favorable impact in diverse chronic diseases of the elderly. Obesity, diabetes and aging share common pathophysiological mechanisms, including mitochondrial impairment and dysfunctional eNOS. Here we review the evidences that eNOS-dependent mitochondrial biogenesis and quality control, and possibly the complex interplay among cellular organelles, may be affected by metabolic diseases and the aging processes, contributing to reduce healthspan and lifespan. Drugs or nutrients able to sustain the eNOS-NO generating system might contribute to maintain organelle homeostasis and represent novel preventive and/or therapeutic approaches to chronic age-related diseases.

## Introduction

Nutrition and medical advancements leading to increased lifespan are not adequately translating into improved healthspan—i.e., into increased number of healthy, fully functional years—in modern societies (Valerio et al., 2011). Particularly, the high prevalence of non-communicable diseases and frailty are major concerns in elderly people (Hunter and Reddy, 2013). Globally, the problems are expected to worsen: the World Health Organization estimates that nearly one in four people will be older than 60 by 2050. It is also estimated that approximately one in three adults is obese in Western countries (Ogden et al., 2014). Both aging and obesity are major risk factors for several diseases such as diabetes, heart disease, stroke and certain types of cancers. Gaining wellbeing at all stages of life and maximizing healthy life expectancy are major goals of the post-2015 sustainable agenda (Hunter and Reddy, 2013).

Present-day gerontology research suggests that, unlike traditional approaches that focus on specific diseases, deciphering, and targeting the aging process itself could be the most clever approach toward increased healthspan. Nonetheless, the emerging epidemics of obesity and diabetes (whose prevalence at all ages is growing due to unhealthy habits and economic crisis) can counteract the benefits of reducing chronic disorders throughout the world. Preventing the metabolic consequences of inadequate diet through groundbreaking transdisciplinary investigation should be a primary research focus (Nisoli and Valerio, 2014).

Multiple cell effectors work together to cause the senescent cell phenotype. Particularly, two cellular organelles—nucleus and mitochondrion—have been implicated in the “wear and tear” aspects of aging (Nisoli and Valerio, 2014). Other organelles, including the endoplasmic reticulum (ER), peroxisomes and lysosomes, have been studied in relation to aging mechanisms. Since cellular organelles act in a coordinated manner, we have recently proposed an aging organelle network theory, exploring the possibility that the complex interplay among mitochondria and other cellular organelles may be affected by the aging process (Nisoli and Valerio, 2014). An urgent challenge is to uncover the molecular mechanism(s) regulating the endomembrane systems in cellular aging. Here we review the recent evidence indicating the relevant role played by the nitric oxide (NO) signaling system in the improvement of the age-related metabolic disorders and in healthspan extension. Moreover, we will summarize the current knowledge regarding the role of NO in organelle crosstalk and quality control.

## Healthy effects of calorie restriction and exercise: the role of eNOS and mitochondrial biogenesis

### Nitric oxide, reactive nitrogen species and the hormetic effect

**Figure 1:**
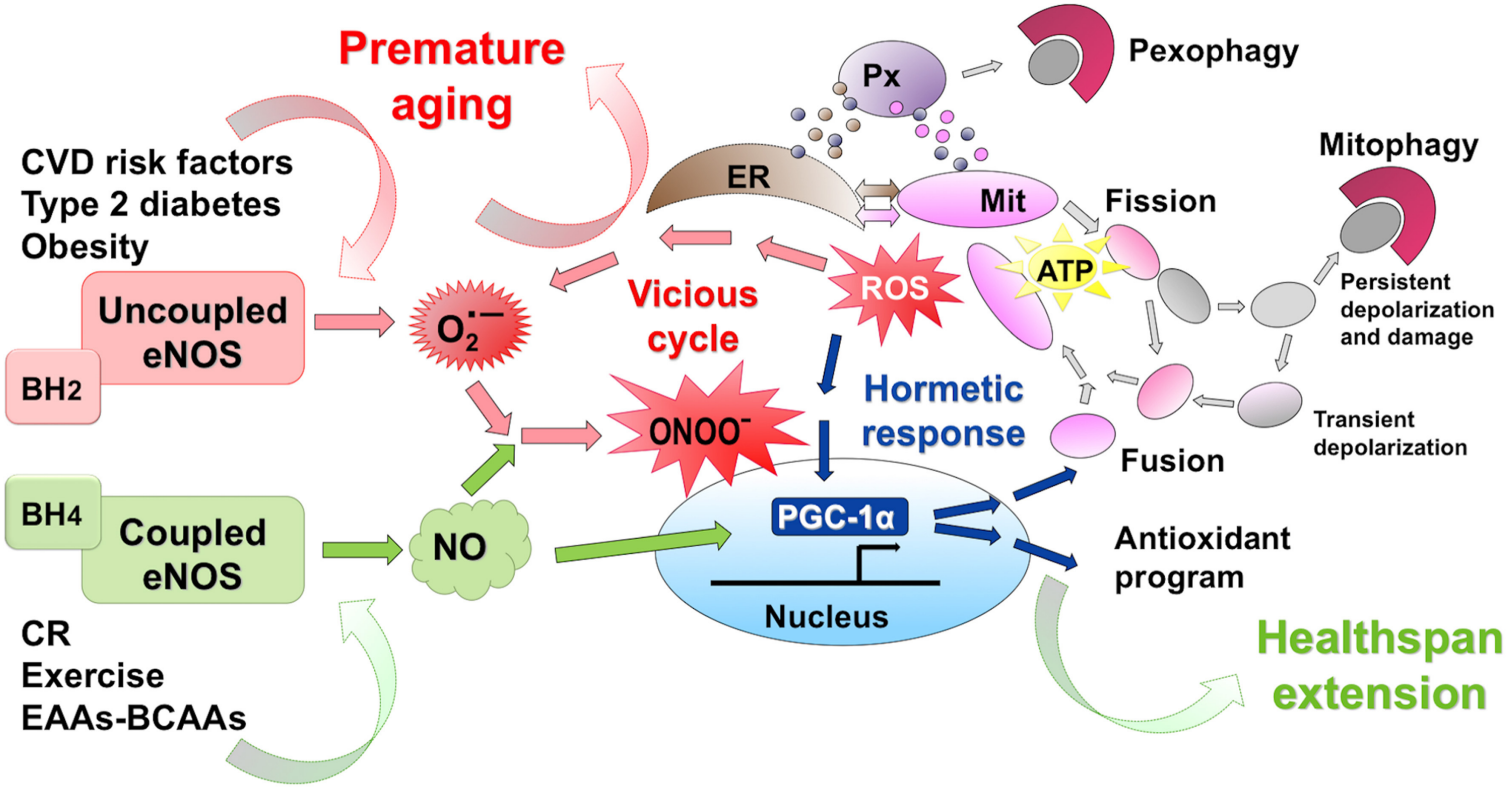
**eNOS activation promotes mithormesis and healthspan extension**. Calorie restriction (CR) and exercise act as slight stressors favoring low-level ROS production. Further, CR, exercise and dietary supplementation with a balanced BCAA-EAA formula enhance eNOS expression and NO production. The eNOS-NO-PGC1α pathway boosts mitochondrial renewal, with efficient energy production, and activates the endogenous antioxidant response. Thus, within a finely tuned cellular range, NO and ROS activate mithormesis, an adaptive stress resistance program promoting cell survival and prolonging healthspan. Conversely, cardiovascular risk factors uncouple eNOS and lead to superoxide (O^•−^_2_) and peroxynitrite (ONOO^−^), a powerful oxidant that initiates a vitious cell-damaging cycle that favors chronic age-related diseases.

NO is highly lipophilic, hyperreactive, diffusible free radical gas (Dudzinski et al., 2006) endowed with a short half-life in biological fluids and acting as a fundamental cell signaling molecule in critical physiological processes (Moncada et al., 1991; Pacher et al., 2007). When reacting with superoxide (O^•−^_2_), NO can give rise to reactive nitrogen species (RNS) which, similarly to reactive oxygen species (ROS) can mediate cellular damage in a wide range of conditions (Pacher et al., 2007) (Figure 1). Traditionally, ROS are represented as unwanted by-products of mitochondrial oxidative phosphorylation and major players in decreased lifespan, based on the Harman's free radical theory of aging (Harman, 2009). With the aim to postpone the aging decay, great efforts have been made to prevent ROS formation by administering high doses of natural or synthetic antioxidant compounds (Ristow and Zarse, 2010). However, several prospective clinical trials designed to demonstrate the health-promoting effects of diverse antioxidants failed, at best showing no benefits (vitamin C and selenium) or even promoting diseases or increasing mortality in humans (beta carotene, vitamin A, and vitamin E) introducing the so-called “antioxidant paradox” (Ristow and Zarse, 2010; Li et al., 2014; Nisoli and Valerio, 2014). Present evidence does not support antioxidant supplements for primary or secondary disease prevention (Bjelakovic et al., 2012). Indeed, the latest findings (in contrast to Harman's original hypothesis) suggest that—within specific concentrations and exposure-time intervals—ROS behave as key signaling molecules potentially involved in lifespan (Kaelin, 2005; Loh et al., 2009; Ristow et al., 2009; Ristow and Zarse, 2010; Ristow and Schmeisser, 2014), possibly by upregulating endogenous antioxidant levels in the human body. The “mitochondrial hormesis” or “mitohormesis” hypothesis suggest that strategies activating low-level stress may indeed favor longevity (Tapia, 2006; Ristow and Schmeisser, 2014) (Figure 1). Lately, supplying weak pro-oxidants, with the aim to promote the composite endogenous antioxidant response, has been suggested as a more useful approach to the treatment and prevention of diseases (Halliwell, 2013).

### Prolongevity mechanisms activated by calorie restriction

In 2003, endogenous NO produced at low doses by endothelial NO synthase (eNOS) was firstly demonstrated to promote mitochondrial biogenesis in several cells and tissues (Nisoli et al., 2003, 2004). Since then, eNOS and downstream NO signaling are appreciated as major metabolic determinants for the regulation of mitochondrial biogenesis (Scarpulla, 2008). The downstream induction of the expression of peroxisome proliferator–activated receptor γ coactivator 1α (PGC-1α) is essential for this effect (Nisoli et al., 2003). It has been established that NO-induced mitochondrial biogenesis requires cyclic guanosine mono-phosphate (cGMP) (Nisoli et al., 2003; Kalogeris et al., 2014). As a consequence of NO-cGMP production, key nuclear-encoded mitochondrial biogenesis transcription factors (including nuclear respiratory factor-1 and mitochondrial transcription factor A) are induced, mitochondrial DNA (mtDNA) levels are increased and mitochondrial function is activated (Nisoli et al., 2003, 2004). Besides this, NO is able to activate PGC-1α via AMPK or in a p53-dependent manner, with a possibility of multiple overlapping pathways (Komen and Thorburn, 2014). Further, NO acting via cGMP induces the expression of the SIRT1 deacetylase (Nisoli et al., 2005) a well-known PGC-1α activity inducer, as discussed below. Clear establishment of the full NO-dependent pathway involved in these phenomena is an interesting point awaiting further research.

Subsequently it has been reported that eNOS expression is induced, and NO-dependent mitochondrial biogenesis is augmented, in the every-other-day feeding model of calorie restriction (CR) in mice (Nisoli et al., 2005). Chronic CR without malnutrition, acting as a low-energy stress condition, is considered the gold standard intervention to extend lifespan in most species. Although the possibility that CR could promote lifespan extension in humans is currently debated, the impact of CR in promoting healthspan by attenuating age-related disease is a consistent finding (Fontana et al., 2010). Calorie-restricted mice had upregulated PGC-1α and sirtuin 1 (SIRT1, the mammalian deacetylase ortholog of the life-extending yeast gene Sir2) and induced endogenous antioxidant response (see also details below) (Nisoli et al., 2005). It should also be mentioned that, although preliminary evidence suggests that high SIRT1 expression is not required for CR-induced lifespan extension (Mercken et al., 2014), its activation extends survival of male mice on a standard diet (Mercken et al., 2013). Interestingly, both SIRT1 and the mitochondrial homologous protein SIRT3, which is also induced by CR, increase eNOS and PGC-1α biological activity through protein deacetylation (Mattagajasingh et al., 2007; Gouspillou and Hepple, 2013; Guarente, 2013). Additionally, CR induces nuclear localization of SIRT1 in a mechanism requiring eNOS, so that SIRT1 and eNOS may comprise a mutually reinforcing activity loop (Guarente, 2013).

Notably, the CR effects were blunted in eNOS-null (eNOS^−/−^) mice (Nisoli et al., 2005), a strain exhibiting metabolic dysfunctions, age-related diseases and shortened lifespan (Valerio et al., 2011). The mitochondrial biogenic effect of CR has been recently challenged in different rodent models (Hancock et al., 2011; Lanza et al., 2012) particularly due to the lack of demonstration of increased mitochondrial protein synthesis rate *in vivo* (Miller et al., 2012). However, evidence from diverse groups support the initial findings, demonstrating that CR increases PGC-1α and the entire paradigmatic transcriptional activation involved in mitochondrial biogenesis, as well as key proteins and/or other relevant markers [including mtDNA abundance and mitochondrial number] in flyes (Zid et al., 2009), rodents (Sreekumar et al., 2002; López-Lluch et al., 2006; Civitarese et al., 2007; Cerqueira et al., 2011) and humans (Civitarese et al., 2007; Mercken et al., 2013). The above reported controversy is likely amplified by virtue of the different CR protocols (percent decrease of *ad libitum* feeding vs. alternate-day-fasting and/or animal species or strain difference), an issue to be further elucidated to fully understand the complexities herein (Gouspillou and Hepple, 2013).

That NO may extend lifespan has been supported by the relevant observations by Li and colleagues (Li et al., 2011). By screening several high-NO yeast mutant strains and selecting the top NO producers, the authors showed a positive correlation between increased NO production, mitochondrial respiration, and longevity. Moreover, treatment with the NO donor S-nitrosoglutathione extended yeast lifespan, in part by increasing stress responses, possibly via inducing a hormetic effect. While CR significantly increases NO production in yeast, some high-NO yeast mutants only partially mimicked CR, suggesting that activation of different subsets of stress response pathway or additional mechanisms contribute to CR-mediated life extension in these mutants. Noteworthy in this respect are the recent observations in *C. elegans*, a roundworm that lacks NOS and compensates for its own deficit of NO production by eating its natural food (bacteria). A recent finding showed that bacterial NO produced inside the worm acts to promote longevity and stress resistance via a defined group of genes that function under the dual control of HSF-1 and DAF-16 transcription factors (Gusarov et al., 2013).

### Healthspan-extending mechanisms of exercise

Regular exercise has a beneficial impact against various disorders typically accompany aging, including diabetes, sarcopenia, and osteoporosis, and fosters cardiovascular health (Fontana et al., 2010). Notwithstanding, exercise intensity, duration, and frequency needs to be controlled in the elderly since excessive exercise increases mortality at older ages (de Cabo et al., 2014). Contrary to CR, which is able to delay aging processes resulting in increased mean and maximum lifespan, exercise fails to extend maximum lifespan (Holloszy, 1998; Mercken et al., 2012). However, exercise and CR are mechanistically related to some extent, since both induce mitochondrial biogenesis and autophagy (He et al., 2012), two strictly interconnected cellular processes which are coregulated to replace old inefficient mitochondria with new, fuel-efficient ones (Michel et al., 2012). Because these two health-promoting interventions also display distinct molecular signatures (Mercken et al., 2013), the integration of CR regimens with regular exercise might result in synergistic healthy effects, as reported measuring insulin sensitivity and circulating C-reactive protein in nonobese subjects (de Cabo et al., 2014).

Among the physiological adaptations induced by regular exercise, a major event is the upregulation of eNOS expression and the consequent increase of tissue NO production, an event also measured in real-time through skeletal muscle microdialysis in exercising human subjects (Hellsten et al., 2007). This in turn induces cell glucose uptake in skeletal and cardiac muscle to meet increased metabolic requirement (Zhang et al., 2007; Lee-Young et al., 2010; Vettor et al., 2013). Two recent studies provides strong evidence that the eNOS-NO-PGC-1α-dependent mitochondrial biogenesis plays a crucial role in the metabolic activation taking place in heart and white adipose tissue in response to physical activity (Vettor et al., 2013; Trevellin et al., 2014). At this point, it is of interest to describe the NO biochemistry and eNOS biology and to review their relevance in health preservation.

## The discovery of NO as a gaseous messenger

As early as 1787, Antoine Lavoisier discovered nitrogen and named it azote (N), meaning without life, due to its lack of chemical reactivity and inability to support life when provided as the atmosphere. However, nitrogen is presents in amino acids and in the bases that form DNA and RNA, and the metabolism of nitrogenous compounds is central to the metabolic processes of all living organisms. Unable to utilize inorganic nitrogen, animals rely on plant foods to acquire nitrogen, and arginine from seed proteins is their major nitrogen source. Free arginine within the body also derives from endogenous synthesis and turnover of body proteins. Arginine is a so-called conditionally essential amino acid for humans, since its synthesis cannot fully meet the needs under certain pathophysiological conditions, including hypercatabolic responses to stress or injury. Apart from its involvement in protein structure and function, arginine has a role as the precursor for the synthesis of NO (Morris, 2006).

Breakthrough studies conducted around the '80s led to the discovery of NO biological actions. Ferid Murad in 1977 discovered the release of NO from nitroglycerine and its action on vascular smooth muscle (Katsuki and Murad, 1977). In 1987, Salvador Moncada and Louis Ignarro independently demonstrated that the endothelium-derived relaxing factor (discovered by Furchgott and Zawadzki, 1980) is NO (Ignarro et al., 1987; Palmer et al., 1987). NO is produced in vary degrees in the cardiovascular, nervous, digestive and immune systems where it modulates a variety of pathophysiological conditions. NO consists of a single oxygen atom bonded to a nitrogen atom through a chemical bond that exhibits partial double bond and partial triple bond character as a result of an unpaired electron (Dudzinski et al., 2006). As a free radical, the NO molecule has unique reactivity that is responsible for its interaction with numerous targets. As a lipophilic gas, NO acts as an unorthodox messenger molecule, which readily diffuses and traverses multiple cell membranes to signal at some distance from its source (Dudzinski et al., 2006).

The effects of NO are mediated by activation of the heme-containing soluble guanylyl cyclase (sGC), with formation of cGMP (Katsuki and Murad, 1977). Studies involving bacterial proteins from the heme-nitric oxide/oxygen (H-NOX) binding family of gas receptors (sharing high sequence homology and conserved residues with the sGC heme pocket) help understanding the mechanism of sGC activation. Crucial to NO-dependent activation of H-NOX proteins is the formation of a five-coordinate NO complex (Herzik et al., 2014). High-resolution crystal structures of a bacterial H-NOX protein in the unligated and NO-bound state show that NO binding leads to a pronounced conformational change in the protein as a result of structural rearrangements in the heme pocket (Herzik et al., 2014). NO can also signal independent of cGMP, by (i) competitively inhibiting cytochrome c oxidase, the terminal heme-containing enzyme in the mitochondrial electron transport chain; (ii) interacting with critical thiols in proteins leading to S-nitrosation, which regulates the function of many proteins; (iii) reacting with superoxide, thus forming RNS and promoting tyrosine nitration of amino acids, which can also affect protein function (Larsen et al., 2012).

## Advances toward a better understanding of eNOS

NO is produced by three NOS isoforms: the neuronal (nNOS, alternatively designated NOS1 as it was the first NOS isoform to be discovered), inducible (iNOS or NOS2), and endothelial (eNOS or NOS3) ones. Names of nNOS and eNOS derived from the tissues in which they were first identified. NOS isoforms differ in their expression, subcellular localizations and mechanistic features, which are responsible for their unique pathophysiological roles. eNOS is classified as a L-arginine, NADPH: oxygen oxidoreductase, utilizing flavins, and tetrahydrobiopterin (BH4) as cofactors and forming homodimers during activation (Förstermann and Sessa, 2012). The enzymatic reaction generating NO involves the transfer of electrons from NADPH, via the flavins in the C-terminal reductase domain, to the heme in the N-terminal oxidase domain, where the substrate L-arginine is oxidized to L-citrulline and NO (Qian and Fulton, 2013). To efficiently produce NO, eNOS must effectively coordinate the binding of multiple substrates and cofactors. Disruption of this highly coordinated catalysis can result in the production of superoxide and peroxynitrite (Dudzinski et al., 2006).

Constitutively expressed in the endothelium, eNOS is responsible for the formation of vascular NO under physiological condition, and is induced by shear stress or chemical stimuli. The enzyme is activated by increased intracellular Ca^2+^, and by a complex integration of transcriptional/post-transcriptional mechanisms and post-translational modifications (serine and tyrosine phosphorylation; S-nitrosylation, acetylation) as well as by substrate and cofactor availability and protein-protein interaction. eNOS is enriched in caveolae (the small invaginations of the plasma membrane, especially abundant in endothelial cells and adipocytes) which may serve as microdomains for optimized NO-sGC-cGMP signaling (Tsai et al., 2012). Further, native eNOS can shuttle between distinct subcellular domains, having been detected in the cytosol, Golgi apparatus, mitochondria, and even in the nucleus of endothelial cells (Feng et al., 1999), brown adipocytes (Giordano et al., 2002) and other cells, paralleling sGC localization (Gobeil et al., 2006). Comprehensive reviews have been published on eNOS and its regulation and we refer readers to those articles for complete information (Dudzinski et al., 2006; Qian and Fulton, 2013).

Well-characterized actions of NO include the stimulation of vasodilation, vessel formation (Ziche et al., 1994), and mitochondrial biogenesis (Nisoli et al., 2003) as well as inhibition of smooth muscle cell proliferation, leukocyte adhesion, platelet aggregation, and mitochondrial respiration (Qian and Fulton, 2013). Thus, thanks to its antihypertensive, antithrombotic and anti-atherosclerotic properties, eNOS-derived NO represents a key molecule in vasoprotection (Förstermann and Li, 2011). Following the original study demonstrating that eNOS-derived NO promotes mitochondrial biogenesis (Nisoli et al., 2003), the eNOS-NO-PGC-1α pathway has been shown to be involved in several health-promoting actions. It has been found to be downregulated in adipose tissue of obese mice and rats (Valerio et al., 2006). Consistently, eNOS overexpression exerts anti-obesogenic effects in high fat-fed mice through improved mitochondrial biogenesis and activity in adipose tissue (Sansbury et al., 2012). The eNOS-NO-PGC-1α pathway is also implicated in anti-aging effects. Besides its activation in calorie-restricted mice (Nisoli et al., 2005) it has an essential role in healthspan extension observed when mice diet is supplemented with a balanced formula of branched-chain amino acids (BCAAs) and other essential amino acids (EAAs) (D'Antona et al., 2010).

Under high oxidative stress in pathological conditions, eNOS may become dysfunctional and contribute to cell damage. In fact, persisting oxidative stress renders eNOS uncoupled (i.e., O_2_ reduction is uncoupled from NO synthesis), such that the enzyme no longer produces the vasoprotective NO, but superoxide (Li et al., 2014). Among the possible mechanisms involved in eNOS uncoupling, depletion of the eNOS cofactor BH4 (due ROS-dependent oxidation) is likely to play the major role. Another cause of eNOS uncoupling is L-arginine deficiency due to increased arginase activity (Li et al., 2014). Uncoupling of eNOS further creates a vicious cycle (Figure 1), since superoxide and subsequent peroxynitrite overproduction enhances BH4 oxidation and upregulates arginase expression/activity. Pre-existing oxidative stress is thus potentiated to the point that it acts as a crucial determinant in cardiovascular disease (Li et al., 2014). This particular issue is becoming an urgent research topic, aimed at identifying therapeutic intervention that optimize eNOS function.

## Relevance of eNOS in human disease and longevity

### eNOS dysfunction and metabolic diseases

Depletion of the eNOS gene induces hyperinsulinemia and insulin resistance (Duplain et al., 2001). A body of evidence also demonstrates that reduced NO bioavailability (as a consequence to multiple-level eNOS dysfunctional regulation) is a key mechanism of endothelial dysfunction. The complex relationship between endothelial dysfunction and insulin resistance in diabetes, obesity and metabolic syndrome has recently been reviewed (Huang, 2009). Mice lacking eNOS fail to respond to cold exposure by thermogenesis, and their brown adipose tissue development and function are defective (Nisoli et al., 2003; Huang, 2009). Abnormalities in energy homeostasis due to reduced eNOS-derived NO increase the risk for cardiometabolic diseases. In fact, eNOS^−/−^ mice are considered a model for the metabolic syndrome (Nisoli et al., 2007) because they combine its main defining features, including hypertension, endothelial dysfunction, insulin resistance and obesity. This phenotype is recapitulated in mice bearing unphosphorylatable eNOS mutations at S1176 (Kashiwagi et al., 2013). Conversely, mice bearing phosphomimetic eNOS mutations at S1176, resulting in increased enzyme activity and NO production, show improved insulin sensitivity and resistance to high fat-induced weight gain (Kashiwagi et al., 2013). Not only eNOS^−/−^ mice have defective mitochondrial biogenesis and function (Nisoli et al., 2003), but also they fail to activate mitochondrial biogenesis in response to cold, CR, exercise or dietary interventions (Nisoli et al., 2005; D'Antona et al., 2010; Vettor et al., 2013; Trevellin et al., 2014). The consequences of reduced NO bioavailability have been explored also in humans. Downregulation of multiple cell signals converging in the eNOS-NO system occurs in type 2 diabetes mellitus (T2DM), and contributes to increase the vulnerability of diabetic patients to atherosclerosis, hypertension, and coronary heart diseases (Sharma and Khanna, 2013).

### eNOS dysfunction in heart failure

A deficiency of mitochondrial energetics has been documented both in cardiac aging and in heart failure. Changes in eNOS have been described in experimental and human cardiac failure with somewhat mixed results (Carnicer et al., 2013). Chronic eNOS deletion has been found to induce age-related concentric hypertrophy (Barouch et al., 2002) and to worsen pathological left ventricular remodeling after myocardial infarction in mice (Scherrer-Crosbie et al., 2001). Consistently, cardiomyocyte-restricted eNOS restoration attenuated compensatory hypertrophy and improved left ventricular performance after myocardial infarction (Janssens et al., 2004) and protected against chronic pressure overload (Buys et al., 2007). However, eNOS^−/−^ mice displayed modest hypertrophy and preserved function compared to wild-type mice after chronic transverse aortic constriction (TAC), a severe condition inducing eNOS uncoupling and increased ROS production (Takimoto et al., 2005). Thus, while eNOS is usually protective against maladaptive stress, it can paradoxically contribute to cardiac pathology in uncoupled conditions (Carnicer et al., 2013). Recent evidence also supports a role for the scaffolding protein Cav-3 in regulating heart NO-sGC-cGMP signaling. A severe depression of NO-dependent activation of sGC has been reported in the TAC mouse model, partly due to sGC oxidation owing to its altered interactions with Cav-3 and modified subcellular compartmentation (Tsai et al., 2012). Interestingly, eNOS-NO signaling contributes to the beneficial effects of statins, angiotensin converting enzyme inhibitors and AT1 receptor blockers in experimental heart failure (Shimokawa and Tsutsui, 2010).

### eNOS allelic variants in human diseases

Among the several eNOS variants so far described, the eNOS D298 and IVS18 + 27C alleles have been found to be significantly associated to T2DM and metabolic syndrome (Monti et al., 2003). In addition, endothelial dysfunction and an atherogenic profile favoring coronary artery disease (CAD) and restenosis after coronary stent implantation were associated with the same eNOS variant (Galluccio et al., 2008). A recent investigation further demonstrates the significant association between eNOS −786C > T polymorphism and CAD (Liu et al., 2014). Interestingly, the −665C > T polymorphism in the eNOS gene predicts increased cardiovascular morbidity and mortality independently of blood pressure and other risk factors in a general population of white Europeans (Olivi et al., 2014). Although to be interpreted with caution, these findings suggest that deficient NO generation as a consequence of eNOS gene variation possibly contributes to the pathogenesis of complex diseases. Further research will be necessary to elucidate the role of these variants in the increased cardiometabolic risk.

### eNOS gene variants in long-living humans

Exceptional longevity in humans is a complex trait. A number of genetic variants are associated in centenarians and long-living individuals. A recent genome-wide association study on 410 long-livings and 553 young controls has identified the rs10491334 gene variant of the Ca^2+^/calmodulin-dependent protein kinase (CAMKIV) (Malovini et al., 2011). Homozygous carriers of rs10491334 have a significant reduction in CAMKIV expression. *In vitro* analysis established that CAMKIV activates the survival proteins AKT, SIRT1, and FOXO3A (Malovini et al., 2011). The authors found a reduced frequency of carriers of the minor allele among centenarians (Malovini et al., 2011). Thus, the results point to a detrimental role for the rs10491334 allele in aging process due to reduced survival genes. Interestingly, the prolongevity genes activated by CAMKIV (that is AKT, SIRT1, and FOXO3A) all can promote eNOS activity (Puca et al., 2012; Xia et al., 2013), thus suggesting that long-living individuals and centenarians are selected among those with high NO production.

## Metabolic diseases and senescence share mitochondrial impairments

A few years ago, we described a marked reduction in eNOS expression, mitochondrial biogenesis and ATP production, together with changes in mitochondrial morphology reminiscent of increased fission dynamics, in fat and skeletal muscles of rodents with genetically- or high fat diet-induced obesity (Valerio et al., 2006). Obesity is increasingly being interpreted as a state of premature aging (Tzanetakou et al., 2012; Nisoli and Valerio, 2014). Notably, NO availability as well as the mitochondrial biogenic capacity are reduced in aged tissues (Nisoli and Valerio, 2014). Application of a novel biomarker of aging has recently revealed a strong correlation between high body mass index and the epigenetic age of liver tissue, with reduced expression of nuclear mitochondrial genes that play a role in oxidative phosphorylation and electron transport (Horvath et al., 2014). Proteomic work lately confirms that obesity parallels key metabolic perturbations observed in the aging process (Tzanetakou et al., 2012; de Castro et al., 2013).

Mitochondrial biology has been extensively investigated in elderly subjects. Aged human skeletal muscles display increased mtDNA mutations. Important evidence that hindering mitochondrial pathology restores health and longevity has been presented by Safdar and colleagues, who studied the progeroid mice which express a mtDNA polymerase-γ variant, and exhibit elevated mtDNA point mutations, systemic mitochondrial dysfunction, multisystem pathology, and reduced lifespan (Safdar et al., 2011). Interestingly, endurance exercise training rejuvenated mice mitochondrial turnover and respiratory capacity, and abolished symptoms of accelerated aging and premature mortality (Safdar et al., 2011).

Recent studies revealed a novel molecular circuit linking nuclear DNA damage to mitochondrial dysfunction in advanced age, and resulting in organ decline and disease development. Genetically-induced progressive telomere shortening in mice induced p53 activation, which directly repressed PGC-1α and PGC-1β. The deficitary mitochondrial biogenesis and function resulted in a premature aging phenotype, with heart failure and liver dysfunction (Sahin et al., 2011). Interestingly, subsequent work demonstrated that deletion of the telomere binding protein RAP1 suppresses PGC-1α leading to obesity and insulin resistance (Duong and Sahin, 2013).

The processes of mitochondrial biogenesis, dynamics, and mitophagy—the so-called mitochondrial lifecycle—represent efficient mechanisms ensuring the health of the cellular pool of mitochondria (see below). Changes in mitochondrial dynamics (increased fission/fragmentation vs. fusion/elongation) progressively reducing bioenergetic efficiency have recently been observed in conditions of excess nutrient intake (Liesa and Shirihai, 2013). Therefore, identification of multiple changes in the mitochondrial lifecycle provide further evidence linking hypernutrition/obesity and senescence through shared organelle dysfunctions, and could explain some of the beneficial effects associated with CR (Liesa and Shirihai, 2013; Nisoli and Valerio, 2014).

Noteworthy, cell-based studies showed that eNOS-derived NO delays age-dependent inhibition of telomerase activity, preventing telomere shortening and counteracting the senescence of endothelial cells (Vasa et al., 2000). Further findings confirmed a hierarchical relationship between eNOS activation and the expression and activity of the catalytic subunit of human telomerase (hTERT) (Vasa et al., 2000; Farsetti et al., 2009). Finally, increased NO bioavailability prevents high glucose-induced endothelial cell senescence (Hayashi et al., 2006), so that eNOS has recently been proposed as an anti-senescence drug target (Hayashi et al., 2014).

## eNOS-derived NO and mitochondria-endoplasmic reticulum connections

The endoplasmic reticulum (ER) is a highly dynamic organelle governing protein post-translational modification, folding, and oligomerization. Disruption of ER homeostasis lead to accumulation of unfolded/misfolded proteins, a condition commonly referred to as “ER stress.” To combat ER stress, cells have evolved a highly conserved adaptive stress response referred to as the unfolded protein response (UPR). Numerous findings have convinced researchers that the ER can undergo ultrastructural and functional changes in aged tissues. ER stress is implicated in age-related disorders, due to the progressive impairment of the UPR and to the deterioration of diverse ER chaperoning systems (Brown and Naidoo, 2012). Molecules able to reduce ER stress, by promoting normal protein folding and/or increasing the unveiling of misfolded proteins, have been proposed to prevent chronic age-related diseases including neurodegenerative diseases, diabetes and atherosclerosis (Brown and Naidoo, 2012).

High-resolution microscopy served to demonstrate that functionally and structurally distinct domains in the ER are sites of membrane contacts with other organelles, where the two membranes are closely apposed without fusing, so that the organelles maintain their distinct identities. Peculiar contacts have been described between the ER and the plasma membrane, mitochondria, Golgi, endosomes and peroxisomes, where a variety of cellular events take place. The functional roles of the stable contact sites between the ER and mitochondria (referred to as mitochondria associated membranes, MAMs) have been reviewed in detail (Rowland and Voeltz, 2012). Among these, Ca^2+^ released by the inositol 1,4,5-triphosphate receptor in the ER is taken up by mitochondria where it is required for efficient bioenergetic functions (Cárdenas et al., 2010). When this Ca^2+^ transfer is deficitary, macroautophagy is activated to sustain cell survival (Cárdenas et al., 2010). Interorganelle contacts also control mitochondrial biogenesis and dynamics. Mitochondrial fission is driven by DRP1, a cytoplasmic protein recruited to the ER-mitochondria contact sites. Mitofusin 2, promoting mitochondrial fusion, tethers contacts between mitochondria and the ER (de Brito and Scorrano, 2008). Finally, live confocal microscopy has demonstrated that tethered ER and mitochondria coordinately move along the cytoskeleton (Rowland and Voeltz, 2012).

Alterations in ER–mitochondria juxtaposition can compromise lipid metabolism, protein synthesis and folding, mitochondrial function and mitochondrial quality control. The possible changes in ER-mitochondria tethering in senescent tissues and their involvement in disease pathogenesis has been so far investigated in the context of neurodegeneration, demonstrating that improvement of the ER-mitochondria crosstalk sustains neuronal bioenergetics and exerts a neuroprotective function (Calì et al., 2013; Nisoli and Valerio, 2014).

The role of eNOS and NO in mediating the ER-mitochondria relationship is just beginning to be explored. Increased NO synthesis by eNOS has been described to induce the ER-mitochondrial chaperone GRP78 and regulate Ca^2+^ fluxes between the mitochondria, the Golgi and the ER, thus conferring organ protection in experimental liver transplantation (Ben Mosbah et al., 2014). It is likely that in the next few years we will witness to exciting developments in this field.

## Peroxisomal biogenesis and connections: a role for NO?

Peroxisomes are remarkably plastic organelles that fulfill important functions including fatty acid oxidation, production of tricarboxylic acid cycle intermediates for mitochondria and control of ROS homeostasis (Schrader et al., 2013). Investigation in recent years has characterized the molecular mechanisms associated with peroxisomal biogenesis. The peroxisome membrane originates from specific ER subdomains or from the growth and division of pre-existing peroxisomes, on the basis of a well-defined dynamic sequence: elongation of the organelle membrane precedes its constriction and final organelle fission (Islinger et al., 2012). Proteins of the PEX11 family act as membrane elongation factors, while dynamin-like proteins dictate fission. Notably, mitochondrial fission proteins such as mitochondrial fission protein 1 (Fis1), mitochondrial fission factor (Mff) and peroxisomal and mitochondrial division factor 1 (PMD1) also localize to peroxisomes. In contrast to mitochondria, however, peroxisomes do not undergo fusion (Islinger et al., 2012). PGC-1α—the master transcriptional co-activator of mitochondrial biogenesis—is also able to increase peroxisome biogenesis (Bagattin et al., 2010). Accordingly, our preliminary experiments show that treatment with the NO donor DETA-NO induces PEX11α and PEX11β expression in differentiated C2C12 myocytes, thus possibly promoting peroxisome proliferation (Nisoli et al., unpublished). All in all, peroxisomes and mitochondria share key components of their division machineries, and are rejuvenated by common signals and transcriptional pathways (Nisoli and Valerio, 2014).

Peroxisomes and mitochondria interact each other and communicate with other subcellular compartments (Camões et al., 2009). Studies in yeast suggest that protein import into the peroxisome modulates this interplay (Beach et al., 2012). With replicative and chronological aging, the decline in the efficiency of peroxisomal protein import initiates a pro-aging spiral within this endomembrane system (Titorenko and Terlecky, 2011). Though our understanding of how peroxisomes are incorporated into metabolic pathways is just beginning to emerge, the existence of a peroxisome-mitochondria network suggest possible implications for human diseases. Indeed, both organelles are supposed to contribute to age-related pathological conditions including ischemia-reperfusion injury, cancer, T2DM, and neurodegenerative diseases (Camões et al., 2009), in which NO does play relevant roles.

## Organelle quality control is affected by aging

Autophagy is a non-specific cellular degradative pathway conserved in eukaryotes to assure intracellular organelle quality. Mitochondrial homeostasis is maintained by several quality-control mechanisms, including the coordinated events of biogenesis, autophagy and the mitochondrial UPR (UPRmt) (Liesa and Shirihai, 2013; Nisoli and Valerio, 2014).

The selective degradation mechanism for mitochondria is mitophagy. The mitochondrial quality-control system is mostly based on fission events, which produce mitochondria with different bioenergetic states. While efficient mitochondria with high membrane potential are reconnected within the functional mitochondrial network through the fusion process, depolarized mitochondria are destroyed by mitophagy (Liesa and Shirihai, 2013). Defects in mitophagy are thought to contribute to age-related pathology, such as Parkinson's disease (Youle and Narendra, 2011).

The mitochondrial form of the Lon protease (mLon) is essential for metabolizing oxidatively damaged mitochondrial proteins. Its peroxisomal counterpart (pLon) plays a role in eliminating damaged peroxisomal proteins. Expression and activity of the Lon proteases declines with age (Titorenko and Terlecky, 2011). Besides being degraded through non-specific autophagy, peroxisomes undergo a specific autophagic process called pexophagy. A global decrease in lysosomal autophagy and a decline in the processes involved in mitochondrial and peroxisomal quality control have been observed in aged tissues (Beach et al., 2012; Settembre et al., 2013). Dysfunctional quality control results in the accumulation of mitochondria with altered morphology and/or membrane potential, whose reduced bioenergetic efficiency heavily contributes to the cumulative damage associated with aging and metabolic diseases (Nisoli and Valerio, 2014).

Indeed, when autophagic mechanism are unable to meet the demand for clearance of damaged intracellular organelles, cell, and organ pathology emerge. In yeast, worms, flies, and mammals, perturbation of autophagy contributes to accumulation of protein aggregates and dysfunctional mitochondria, and takes part to the pathogenesis of various age-dependent chronic diseases resulting in decreased lifespan (Allison et al., 2014). Interestingly, SIRT1-mediated activation of autophagy is essential for lifespan extension by CR in worms (Allison et al., 2014).

The role of NO and its downstream signals in autophagy induction has long been a controversial issue, with studies describing NO as a suppressor of starvation-induced autophagy (Sarkar et al., 2011), or indicating NO-based signaling as an inducer of LPS-mediated autophagy (Yuan et al., 2009). While NOS inhibition altered mitochondrial dynamics with decreased fusion and increased fission, this treatment was found to exert minimal effects on autophagy in the vasculature (Miller et al., 2013). A recent study demonstrated the role of NO as an autophagy inducer via its downstream mediator, 8-nitro-cGMP, in inflammatory conditions (Ito et al., 2013). Interestingly, eNOS-derived NO stabilizes the autophagy-related protein unc-51 like kinase (ULK1) and negatively regulated 26S proteasome functionality (Xing et al., 2014). The NO-mediated increase of ULK1 has been recently described as a novel pathway leading to SIRT1 protein stabilization, though this seems to occur independently of autophagy in endothelial cells (Xing et al., 2014). Nonetheless, given the important implications of autophagy in various diseases of aging (Allison et al., 2014), the role of NO signaling in different types of autophagy (ULK1-dependent or -independent) deserves further investigation.

## Conclusions and future perspectives

Much progress has been made in clarifying the mechanisms regulating mitochondrial lifecycle, mitochondrial-organelle communications and organelle quality system. Future challenge is to deeply investigate the endomembrane system changes in aging and metabolic diseases and the contribution of eNOS-derived NO herein. After more than 30 years since its discovery, we are still finding unsuspected evidence on the pathophysiological roles of NO signaling. In particular, eNOS dysregulation takes part to the pathogenesis of complex-trait metabolic and age-related diseases. Strategies to maintain the eNOS-NO-PGC-1α pathway within a physiological range might restore the impaired mitochondrial bioenergetics of advanced age, possibly mimicking the beneficial effects of CR and moderate exercise (Figure 1). Interestingly, dietary supplementation with a BCAA-enriched EAA formula has been demonstrated to restore impaired cardiac and skeletal muscle mitochondrial biogenesis in an eNOS-dependent manner in aged mice (D'Antona et al., 2010). The BCAA-EAA formula, which is known to exert a variety of beneficial effects in human subjects, prevented oxidative damage, enhanced physical endurance and prolonged mean mice survival (D'Antona et al., 2010; Valerio et al., 2011).

Though still in its infancy, research on the role of the eNOS-NO system in the control of cell organelle connections and quality control might reveal exciting avenues for disease treatment in the coming years. The development of novel therapies aiming to preserve eNOS-NO signaling will benefit from the identification of site-specific interaction with the eNOS structure. Efforts to identify druggable eNOS sites are ongoing (Maron and Michel, 2012). Groundbreaking research is also taking into consideration uncoupled eNOS as a druggable target (Li and Förstermann, 2014). Some drugs in clinical use (including statins and various inhibitors of the renin-angiotensin-aldosterone system) have been shown to prevent eNOS uncoupling under experimental conditions. Since eNOS uncoupling is reversible, it should be of interest to identify conventional drugs or food able to recouple eNOS, switching it back to a well-functioning, NO producing enzyme. The eNOS transcription enhancers AVE9488 and AVE3085 are promising molecules also acting as eNOS recouplers (Li and Förstermann, 2014). Resveratrol can reverse eNOS uncoupling by increasing BH4 producing enzyme, but has poor bioavailability (Li and Förstermann, 2014). Small molecules acting as specific SIRT1-activating compounds (the so-called STACs) possibly intervening in the SIRT1-eNOS activity loop (Guarente, 2013) also deserve to be investigated in this context. Although our knowledge about the therapeutical usability of the proposed eNOS-targeting molecules in the long-term is limited, further research might provide the market with novel pharmaceuticals or nutraceuticals with fundamental health-promoting effects.

### Conflict of interest statement

The authors declare that the research was conducted in the absence of any commercial or financial relationships that could be construed as a potential conflict of interest.
